# Ill-Behaved Patients

**Published:** 1898-12-10

**Authors:** 


### Editor's Letter-Box.

[Our Correspondents are reminded that prolixity is a great bar to publication, and that brevity of style and conciseness of statement greatly facilitate early insertion.]

### ILL-BEHAVED PATIENTS.

The Hon. Sydney Holland, Chairman of the London Hospital, writes: What joy there is in the Press if hospital authorities make any mistake. "Scandal at a Hospital" is placarded all over the town at once. This is the greatest tribute I know of to the good management of hospitals. Everybody likes to see a saint fall, the better the saint the greater outward moaning, but inward rejoicing, at his backsliding. It is so comforting to feel that other people are as bad as ourselves. If "scandals" at hospitals were frequent there would be no "fat" in them for the penny-a-liners. Once there was a "scandal at the Poplar Hospital," and the inquest was adjourned (I do not mean that this has been the only one; they are perennial at all hospitals). I wrote round to every London paper asking them to send representatives to hear our side of the question at the adjourned inquest, and by this action earned the undying hostility of the poor man who generally reported the inquests. "You have taken five pounds out of my pocket by doing this," he pathetically exclaimed, "as I should have sent reports to all the papers and been paid for them." This from the man who a week before had, I suppose, earned another £5 by sending the report of "scandalous treatment of a patient; murdered before his mother's eyes at the Poplar Hospital." Thenceforward my eyes were opened. As to the latest "scandal at the London Hospital," I will not weary your readers by entering into details to explain why we were not so bad as we were painted. I wish merely to correct an error into which The Hospital has fallen. You write: "In this case the patient was turned out of the London Hospital on Saturday, November 5th, for gross indecency, but was so ill that he died on the Monday following, and we cannot but feel that it was a grave error of judgment merely to put a man out into the street when he was so ill, as this man must have been." I think we might reasonably expect accuracy in attack from The Hospital, whatever we may expect from the *Sun*, *Star*, &c. The man did not die on the "Monday following," but on the Monday week following—nine days after he had been turned out. I admit that it was a wonder he did not die the day he was turned out, not because he was ill enough to have been in any danger, but because if ever a hospital makes a mistake about a patient he always does die, and generally at once and in a most inconveniently public manner. This man showed no symptoms which could have led anyone to think that he was dying, nor was he when he was turned out. May I also add that his filthy lewdness was not the result of delirium at all, but was wilful, and he took good care to control his language and conduct when the house physician was present.

*** The following is the evidence given at the inquest by Police-constable Bell as reported in the press: "Police-constable Bell stated that he found the defendant lying on the footway surrounded by a crowd. He appeared ill and helpless, and some of the persons standing round said he had been turned out of the hospital. Witness conveyed the man to the workhouse infirmary, where he remained until his death on the following Monday."—Ed. *T. H.*

